# A candidate gonococcal outer membrane vesicle vaccine which induces persistent immunity in three genetically distinct species of mouse and is similarly immunogenic when formulated with and without aluminium adjuvant

**DOI:** 10.3389/fimmu.2026.1923399

**Published:** 2026-10-02

**Authors:** Ilaria Onofrio, Thomas Belcher, Molly E. Quinn, Emma Lupoli, Tyler Freslov, Natasha Owen, Senuri N. Dissanayake, Lukasz A. Grudzein, Paula Freixeiro, Christine S. Rollier, Calman A. MacLennan

**Affiliations:** 1 Nuffield Department of Medicine, Jenner Institute, University of Oxford, Oxford, United Kingdom; 2 School of Life Sciences, Keele University, Keele, United Kingdom; 3 School of Biosciences, University of Surrey, Guildford, United Kingdom; 4 Department of Infectious Diseases, Faculty of Medicine, Imperial College London, London, United Kingdom; 5 Department of Immunology and Immunotherapy, College of Medicine and Health, University of Birmingham, Birmingham, United Kingdom

**Keywords:** aluminium hydroxide, gonococcal, gonorrhoea, immune response, longevity, mouse strains, outer membrane vesicle, vaccine

## Abstract

**Background:**

The escalating global incidence of multidrug-resistant *Neisseria gonorrhoeae* necessitates an effective vaccine. While the meningococcal 4CMenB vaccine offers some cross-protection against gonorrhoea, this protection is limited highlighting the need for a targeted gonococcal vaccine. In this study, we evaluate the durability of immune response and characterise the immunogenicity induced by GonoVac, a native Outer Membrane Vesicle (nOMV) vaccine produced from *N. gonorrhoeae* GC_0817560*lpxL1^-^*, across genetically distinct strains of mouse.

**Methods:**

We evaluated the longevity of the immune response induced by two and three doses of GonoVac formulated with aluminium hydroxide (Al(OH)_3_) over a one-year period (49 weeks for C57BL/6 and CD1 mice, 33 weeks for BALB/c mice), compared to the meningococcal group B vaccine, 4CMenB. Identical immunisation schedules were followed in BALB/c, C57BL/6, and CD1 mice. Separately, we examined the impact of Al(OH)_3_ on GonoVac immunogenicity in all three mouse strains. Serological, mucosal, and cellular responses were assessed using ELISA, serum bactericidal assays (SBA) and ELISpot.

**Results:**

GonoVac with Al(OH)_3_ induced robust, long-lasting anti-gonococcal IgG responses in all three strains of mice and significantly higher levels of antibody-dependent complement-mediated killing of gonococci in C57BL/6 and CD1 mice compared with 4CMenB. Immune responses were maintained for up to one year. Serum anti-gonococcal IgG subclass distributions varied across mouse strains and between GonoVac and 4CMenB in the same strain of mouse. GonoVac formulated with and without Al(OH)_3_ elicited high levels of antibody-dependent complement-mediated killing of *N. gonorrhoeae* irrespective of mouse strain. While mucosal IgG responses were robust with both GonoVac formulations, Al(OH)_3_ significantly enhancing cellular immunity in BALB/c and CD1 mice compared with C57BL/6 mice.

**Conclusions:**

Our findings demonstrate that GonoVac, a preclinical candidate gonococcal nOMV vaccine, induces durable and functional anti-gonococcal immune responses that are higher than 4CMenB, across three commonly used laboratory strains of mice.

## 1 Introduction

Gonorrhoea, caused by the Gram-negative bacterium *Neisseria gonorrhoeae*, is the second most common sexually transmitted infection (STI), with over 82 million new cases estimated annually (1, 2). The disease disproportionately affects women and individuals in low- and middle-income countries (LMICs), where limited access to diagnostic facilities, effective treatment, and surveillance systems often results in asymptomatic infections going undetected and untreated (3). Left unmanaged, gonorrhoea can lead to serious sexual and reproductive health complications including pelvic inflammatory disease (PID), infertility, ectopic pregnancy, and increased susceptibility to HIV infection (4, 5). The growing global threat of antimicrobial-resistant *N. gonorrhoeae* has intensified concerns regarding future treatment failure, prompting the WHO to designate *N. gonorrhoeae* as a high priority pathogen and call for urgent development of new interventions, including vaccines (6–9).

Recent clinical observational studies have sparked interest in outer membrane vesicle (OMV)-based vaccine platforms, with serogroup B meningococcal OMV vaccines, such as MeNZB and 4CMenB (Bexsero, GSK), demonstrating moderate cross-protection against gonorrhoea (10, 11). This protection, estimated at 33-46% (12–15), is attributed to substantial antigenic similarity between *N. meningitidis* and *N. gonorrhoeae*, especially among conserved outer membrane proteins (16). In response to these clinical findings, the United Kingdom recently introduced targeted use of 4CMenB for gonorrhoea prevention among gay, bisexual, and other men who have sex with men, populations disproportionately affected by gonococcal infection (17).

However, no established correlate of protection exists for gonorrhoea, making early gonococcal vaccine evaluation particularly challenging. While serum bactericidal assays (SBA), evaluating antibody-dependent complement-mediated killing *in vitro*, are the gold standard recognised correlate of protection for meningococcal disease (18), they remain an uncertain marker in relation to protection against gonorrhoea. Nevertheless, complement-mediated killing is likely to play a role in protection, as individuals with terminal complement deficiencies (C5b-C9), unable to form the Membrane Attack Complex (MAC), as well as those with C2 deficiency, have increased susceptibility to disseminated gonococcal infection (DGI) (19–22).

Building on this foundation, we developed GonoVac, a native OMV (nOMV) vaccine derived from a genetically modified strain of *N. gonorrhoeae*, GC_0817560*lpxL1^-^*, designed to reduce reactogenicity while preserving key immunogenic components (23, 24). GonoVac induces robust immunogenicity in BALB/c mice, including strong systemic and mucosal IgG responses, immunoglobulin class switching, and high levels of antibody-dependent complement-mediated killing (24). As with 4CMenB, GonoVac induces relatively low amounts of serum anti-gonococcal IgA and negligible mucosal anti-gonococcal IgA (24). Nevertheless, we have demonstrated that gonococcal nOMV candidate vaccines accelerate clearance of gonococcal infection in the β-oestradiol mouse model compared with 4CMenB (23).

Most early-stage vaccine studies are conducted in a single inbred mouse strain, often BALB/c mice, which are associated with a Th2 immune phenotype (25–28). This limited scope may not fully capture the immunological variability seen in humans. Indeed, differences in vaccine-induced immune magnitude, kinetics, and durability between inbred and outbred strains of mouse can significantly influence translational relevance. Such variability in vaccine responses due to host genetic factors is well-documented for other pathogens and vaccines, including those against influenza, hepatitis B, and measles, where genetic polymorphisms can influence antibody levels, duration of immunity, and occurrence of non-responsiveness (29–31). While BALB/c mice are often used for investigating humoral immune responses, C57BL/6 mice are associated with Th1 immunity and often used for assessing T cell responses (25, 32–34). Outbred mouse strains, such as CD1, more closely mimic human genetic diversity, offering an additional layer of insight into vaccine responsiveness (35, 36).

Durability of immunity is another critical consideration for a gonococcal vaccine. As vaccination strategies are likely to target adolescents prior to sexual debut, protective immune responses must persist for many years to provide meaningful public health benefit (37), and may vary across different genetic backgrounds. Available clinical effectiveness data suggest that the OMV in the 4CMenB vaccine offer limited duration of protection against gonorrhoea, although interpretation is limited due to reduced power when splitting subjects according to time since vaccination (10).

In this study, we investigated two key aspects of the immune response induced by the GonoVac candidate vaccine in three genetically distinct strains of mouse. First, we evaluated the durability of systemic antibody responses to GonoVac formulated with Al(OH)_3_ over a one-year period (49 weeks for C57BL/6 and CD1 mice, 33 weeks for BALB/c mice), including anti-gonococcal IgG titres, subclass distribution, avidity, and SBA titres. The licensed 4CMenB vaccine was used as a comparator, given its partial effectiveness against gonorrhoea in humans and in the murine model against gonococcal lower genital tract infection (12, 13, 15, 23, 38).

Second, we characterised the immunogenicity of GonoVac formulated with and without Al(OH)_3_, by assessing both humoral responses and cellular immunity (IFN-γ-, IL-4-, IL-17A-producing cells), across three strains of mouse. Therefore, this study collectively evaluates the breadth and persistence of GonoVac-induced immunity, addressing inter-strain variability to support further translation to human clinical trials.

## 2 Materials and methods

### 2.1 Strains and growth of *N. gonorrhoeae*

*N. gonorrhoeae* GC_0817560 used in this study was obtained from the WHO Collaborating Centre for Gonorrhoea and other Sexually Transmitted Infections (Örebro, Sweden). As previously described, the strain was genetically modified to attenuate endotoxin activity by deleting the *lpxL1* gene for GonoVac nOMV production (23).

Bacteria were cultured from frozen glycerol stocks on gonococcal agar (GCA) plates supplemented with 2% Vitox (Oxoid, Becton Dickinson, UK), and incubated overnight at 37 °C in 5% CO_2_ (24). For liquid culture, colonies were inoculated into gonococcal broth composed of 15 g/L protease peptone no. 3, 4 g/L K_2_HPO_4_, 1 g/L KH_2_PO_4_, and 5 g/L NaCl (pH 7.2). The medium was supplemented with 1% Kellogg’s supplement I (containing 400 g/L D-glucose, 10 g/L L-glutamine, and 0.002% w/v thiamine pyrophosphate, filter-sterilised using a 0.2 µm filter) (39). Cultures were incubated at 37 °C with 5% CO_2_ under shaking conditions (170 rpm).

### 2.2 nOMV production

GonoVac nOMV were prepared in shake-flasks and bioreactors as previously described (23, 24). nOMV were formulated in phosphate-buffered saline (PBS) and sterile-filtered. Total protein content was determined by BCA assay (Thermo Scientific). A brief proteomic summary of GonoVac *(N. gonorrhoeae* GC_0817560*lpxL1^-^* nOMV) has previously been described (23).

### 2.3 Gonococcal lysate preparation

Lysates for ELISA and cellular assays were prepared from *N. gonorrhoeae* GC_0817560 cultures via mechanical disruption (FastPrep-24) and debris removal by centrifugation and filtration, as previously reported (24).

### 2.4 Animals and immunisation

All animal procedures received approval from the University of Oxford Animal Welfare and Ethical Review Body and were conducted under UK Home Office licence PP2250582 (Study 1) and PP7685090 (Study 2). Female 6–8-week-old inbred BALB/c and C57BL/6, and outbred CD1 mice (Envigo, UK) were immunised intramuscularly in the tibialis muscle (50 μL total per leg) under light isoflurane anaesthesia. Each vaccine dose contained 5 μg GonoVac in PBS, with or without Al(OH)_3_ (Croda). Intermediate blood samples were collected via tail bleed, while terminal samples were obtained by cardiac puncture. Serum was separated after clotting by two cycles of centrifugations (13, 000 x *g* for 10 min) and stored at -40 °C. Vaginal washes were collected by flushing the vaginal canal three times with 30 μL of PBS each time, giving a total volume of 90 μL.

### 2.5 ELISA for serum and mucosal antibodies

ELISA to quantify total IgG, IgM, IgG isotypes (IgG1, IgG2a, IgG2b, IgG3), and mucosal IgG against *N. gonorrhoeae* GC_0817560 lysate were carried out as previously described (24). Briefly, Nunc-Immuno Maxisorp 96-well plates were coated overnight with 6 μg/mL gonococcal lysate in PBS. Plates were blocked with 1% Bovine Serum Albumin (BSA, Sigma Aldrich) in PBS, followed by incubation with serially diluted test samples or standard curves (pooled sera from gonococcal OMV-immunised animals). HRP-conjugated goat anti-mouse secondary antibodies were applied (total IgG: 1:10, 000 Jackson ImmunoResearch; IgG isotypes/IgM: 1:8, 000 SouthernBiotech). Plates were developed using 3, 3′, 5, 5′-Tetramethylbenzidine (TMB) substrate (MP Biomedicals). The colorimetric reaction was stopped with 2 M H_2_SO_4_, and absorbance read at 630 and 450 nm using BioTek microplate reader. Antibody concentrations, expressed in Arbitrary ELISA Units (AEU), were interpolated using a 4-parameter logistic (4PL) model in Gen5 software and normalised to a 1:100 dilution factor for serum. The lower limit of quantification/detection was defined as the end of the linear range of the standard curve, corresponding to 1 AEU multiplied by the minimum dilution factor of 100, i.e. 100 AEU. Accordingly, samples with a titre of ≤100 AEU were considered below the detection limit and reported as 100 AEU. Values below this threshold were not treated as quantifiable measurements.

### 2.6 Avidity ELISA

Anti-gonococcal IgG avidity was assessed by a urea-elution modified ELISA (24). Each serum sample was plated at a single dilution in duplicate on two separate halves of an ELISA plate. After initial binding and washes, one half of the plate was treated with 50 μL/well of 6 M urea (Sigma Aldrich) in PBS for 30 minutes at room temperature, while the other half, which also contained controls and standard curve serum dilutions, was incubated with PBS. The above ELISA protocol was then followed. Avidity Index (AI) was calculated as: AI (%) = (IgG titre with urea/IgG titre without urea) × 100.

### 2.7 Serum bactericidal assay

SBA was performed as described previously (24). Briefly, 20 μL of serially diluted heat-inactivated mouse sera in bactericidal buffer (BB; Hank’s Balanced Salt Solution (HBSS; Gibco) containing 0.5% BSA (Sigma-Aldrich)) was incubated with 10 μL *N. gonorrhoeae* GC_0817560 (6 x 10^4^ CFU/mL). A 10 μL aliquot of pooled human IgM/IgG/IgA-depleted human serum (Pel-freez), serving as an external complement source (previously diluted 1:2 in BB, 12.5% final assay concentration), was then added. Reactions were incubated for 45 min at 37 °C with shaking. Live bacterial cells were enumerated by plating onto GC agar. Bactericidal titres were defined as the highest serum dilution yielding ≥50% killing compared to complement-only controls.

### 2.8 Cellular immune responses

Vaccine-induced cellular responses were quantified by FluoroSpot assays (Mabtech, FSP-4144-10) detecting IFN-γ-, IL-4-, and IL-17A-secreting cells, as previously reported (24). Spleens were harvested and processed into single-cell suspensions using gentleMACS Dissociators and C tubes (Miltenyi Biotec). Cells were treated with RBC lysis buffer, washed, filtered, and resuspended in R10 media (RPMI (Gibco) with 10% of heat-inactivated Foetal Calf Serum (FCS) (Sigma-Aldrich)), 1% L-glutamine (Sigma-Aldrich), 1% Penicillin-Streptomycin (pen/strep; Sigma-Aldrich). Viable cells were counted using a Countess II FL Automated Cell Counter. Cells (2.5 x 10^6^ cells/mL) were added to FluoroSpot plates pre-coated with anti-IFN-γ, anti-IL4, and anti-IL17A monoclonal antibodies. *N. gonorrhoeae* GC_0817560 lysates (75 μg/mL final concentration) with 0.2 µg/mL CD28 as co-stimulus were used for antigen-specific stimulation in triplicate wells. Positive control wells (1 well per sample) contained 25 ng/mL of Phorbol 12-myristate 13-acetate (PMA, Cayman Chemical) and 500 ng/mL of ionomycin (IO, Sigma-Aldrich) (final concentrations). Negative control wells contained unstimulated cells and media only. Cells were stimulated for 24 h, and spots were detected according to manufacturer’s instructions and counted using AID ELISpot software 8.0. Results were averaged from triplicate wells and the unstimulated background control was subtracted.

### 2.9 Statistical analysis

Statistical analyses were performed using GraphPad Prism 10. Data normality was assessed before selecting parametric (ANOVA) or non-parametric (Kruskal–Wallis) tests, followed by appropriate *post hoc* analyses (Tukey’s or Dunn’s test). Two-way ANOVA with Šidák’s correction was used for investigations involving two factors. Antibody ELISA and SBA data were logarithmically transformed (log10 and log2, respectively) prior to two-way ANOVA to minimise variability.

## 3 Results

### 3.1 GonoVac is highly immunogenic in three genetically distinct strains of mouse with serum anti-gonococcal IgG levels that persist unchanged over a year duration in two mouse strains

The persistence of the immune response to GonoVac was assessed over a one-year period in three strains of mouse, BALB/c, C57BL/6 and CD1. Ten 6-8-week-old female mice per group were immunised with either two or three doses of GonoVac, produced in shake flasks and formulated with Al(OH)_3_ (5 µg GonoVac OMV protein-equivalent/259.5µg Al^3+^) in PBS, or 5 µg 4CMenB (OMV protein-equivalent) and administered intramuscularly. CD1 and C57BL/6 were monitored for 49 weeks post-first immunisation, while BALB/c mice were sacrificed at 33 weeks post-first immunisation due to the development of tumours with age. As some mice were lost after reaching humane endpoints due to the length of the study, a subset of 5 to 6 randomly selected mice per group was used for the immunogenicity assessment. Serum samples collected at 9 weeks and at terminal time points (33–49 weeks) were analysed to assess anti-gonococcal antibody responses (**Figure 1A**). This analysis aimed to determine (i) the immune response to GonoVac across different mouse strains, (ii) how the response compared with 4CMenB, and (iii) the durability of the immune response over time.

**Figure 1:**
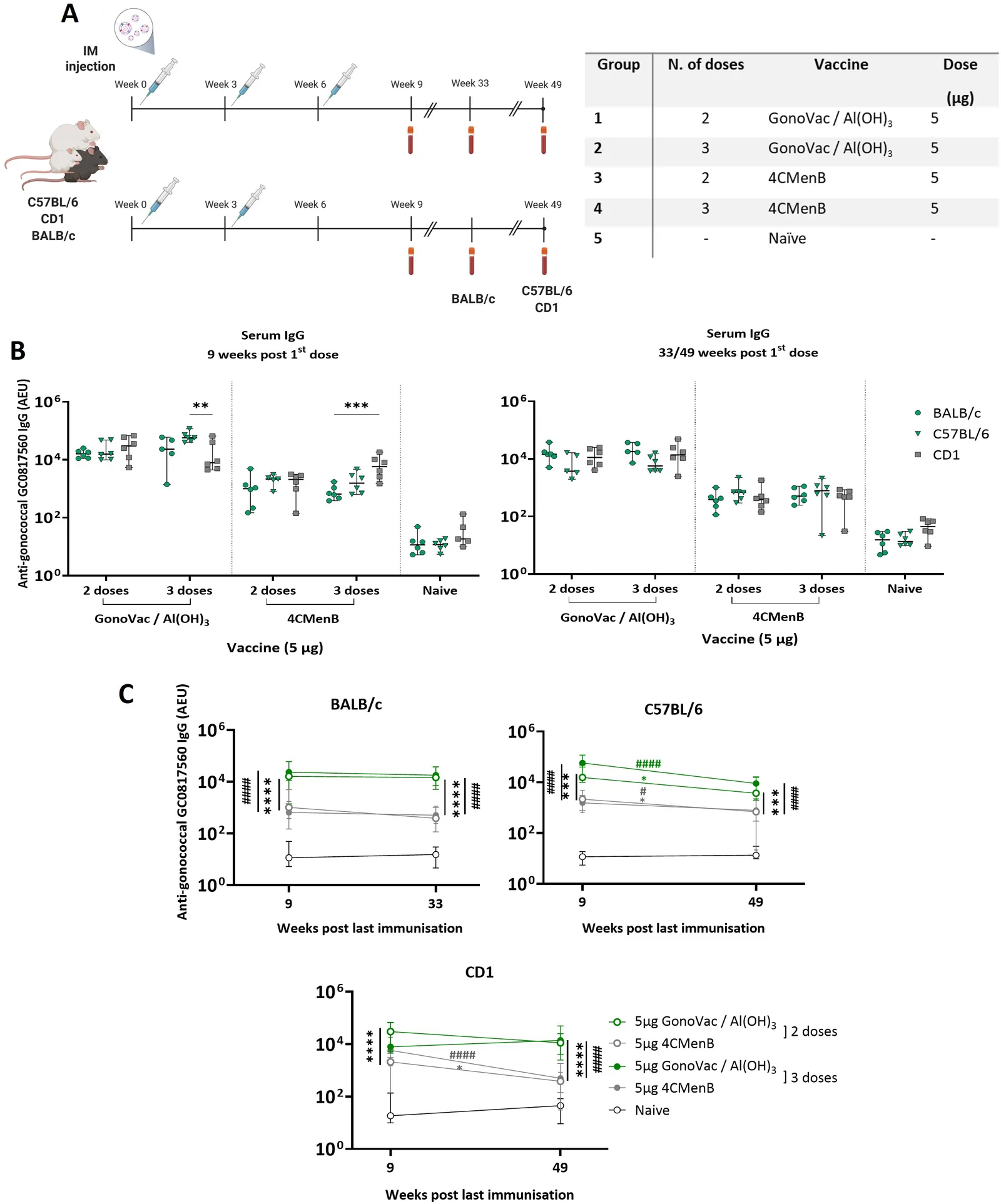
Persistence of the serum anti-gonococcal IgG responses induced by 2 or 3 doses of GonoVac/Al(OH)_3_ or 4CMenB in C57BL/6, BALB/c, and CD1 mice. **(A)** Immunisation schedule: 6-8-week-old female mice were immunised intramuscularly at weeks 0 and 3 or 0, 3 and 6 with 5 µg of flask-produced GonoVac/Al(OH)_3_ or 5 µg of 4CMenB (OMV protein-equivalent) Figure made with https://www.biorender.com/. **(B)** Serum anti-gonococcal IgG responses were measured by ELISA with *N. gonorrhoeae* GC_0817560 lysate as antigen. Responses were assessed at week 9 and at the end of the study (week 49 or 33) in all strains of mouse. Statistical test: 2-way ANOVA with Šídák’s correction for multiple comparisons. P values: **p<0.01, ***p<0.001. Individual mice are represented by circles. Horizontal bars signify medians ± 95% CI. n=5–6 mice/group. **(C)** Persistence of the serum anti-gonococcal IgG response between week 9 and week 33/49 post first immunisation. Statistical tests: 2-way ANOVA with Šídák’s correction for multiple comparisons. Circles represent medians ± 95% CI. n=5–6 mice/group. Significant differences between week 9 and terminal bleed IgG titres are indicated by colour-coded symbols corresponding to each vaccine group:. Significant differences between GonoVac- and 4CMenB-induced IgG responses are indicated at the sides of the graphs. 2-dose regimen, *; 3-dose regimen, #.*/# p<0.05, **/## p<0.01, ***/### p < 0.001, ****/#### p < 0.0001. AEU, Arbitrary ELISA Units.

Minimal inter-strain differences emerged when comparing serum anti-gonococcal IgG levels induced by two immunisations of either GonoVac or 4CMenB (**Figures 1B, C**). Significant differences between mouse strains were apparent only at 9 weeks post-initial dose (6 and 3 weeks after the second or third immunisation respectively), with C57BL/6 mice immunised with three doses of GonoVac having higher serum anti-gonococcal IgG levels compared with CD1 mice (p = 0.009), while CD1 mice that received three 4CMenB immunisations exhibited higher anti-gonococcal IgG than BALB/c (p = 0.0007 **Figure 1B**). At both timepoints, no significant differences were found in anti-gonococcal serum IgG levels between groups receiving two compared with three doses of either vaccine (p≥0.999; **Figure 1B**).

GonoVac induced significantly higher anti-gonococcal IgG titres than naïve controls at all time points across all mouse strains (p<0.0001; **Figure 1B**). At week 9, IgG responses to GonoVac significantly exceeded those of 4CMenB in BALB/c and C57BL/6 mice, regardless of dose (2 doses p ≤ 0.0001; 3 doses p<0.0001), and in CD1 mice after two doses (p<0.0001; **Figure 1C**). By the end of the study, GonoVac-induced IgG were significantly higher than 4CMenB in all strains regardless of the dosing regimen (**Figure 1C**).

Serum anti-gonococcal IgG responses induced by GonoVac and 4CMenB were persistent for the whole duration of the study in BALB/c, but declined significantly over time in C57BL/6 mice with both vaccines. In CD1 mice, anti-gonococcal IgG responses persisted unchanged from 9 to 49 weeks with GonoVac but decreased significantly over that period with 4CMenB. In this strain of mouse, the median of the serum anti-gonococcal IgG titres elicited by 4CMenB decreased by approximately half a log_10_ unit (2 doses: log_10_ 4.19 to 3.57, p = 0.01; 3 doses: log_10_ 4.76 to 3.95, p<0.0001; **Figure 1C**) by the end of the study. Similarly, 4CMenB-induced anti-gonococcal IgG levels were significantly reduced by the study conclusion in C57BL/6 (2 doses: log_10_ 3.33 to 2.84, p = 0.046; 3 doses log_10_ 3.19 to 2.89, p = 0.02; **Figure 1C**) and, unlike GonoVac, in CD1 mice (2 doses: log_10_ 3.32 to 2.58, p = 0.04; log_10_ 3.76 to 2.69, 3 doses: p<0.0001; **Figure 1C**).

### 3.2 GonoVac and 4CMenB induce differential serum subclass distributions across different genetic strains of mouse

GonoVac-induced anti-gonococcal serum IgG1 responses were significantly higher in CD1 and BALB/c than in C57BL/6 mice (p = 0.0009 and p = 0.0005, respectively), and, by contrast, higher in C57BL/6 compared to CD1 and BALB/c mice following immunisation with 4CMenB (p<0.0001; **Figure 2A**). Considering that both vaccine formulations contained Al(OH)_3_, these differential responses suggest that the two vaccines elicit distinct IgG isotype responses depending on the strain of mouse, rather than any adjuvant effect. Baseline anti-gonococcal IgG1 levels in the sera of naïve C57BL/6 mice were also significantly lower than in BALB/c and CD1 mice (p = 0.0007 and p = 0.005).

**Figure 2:**
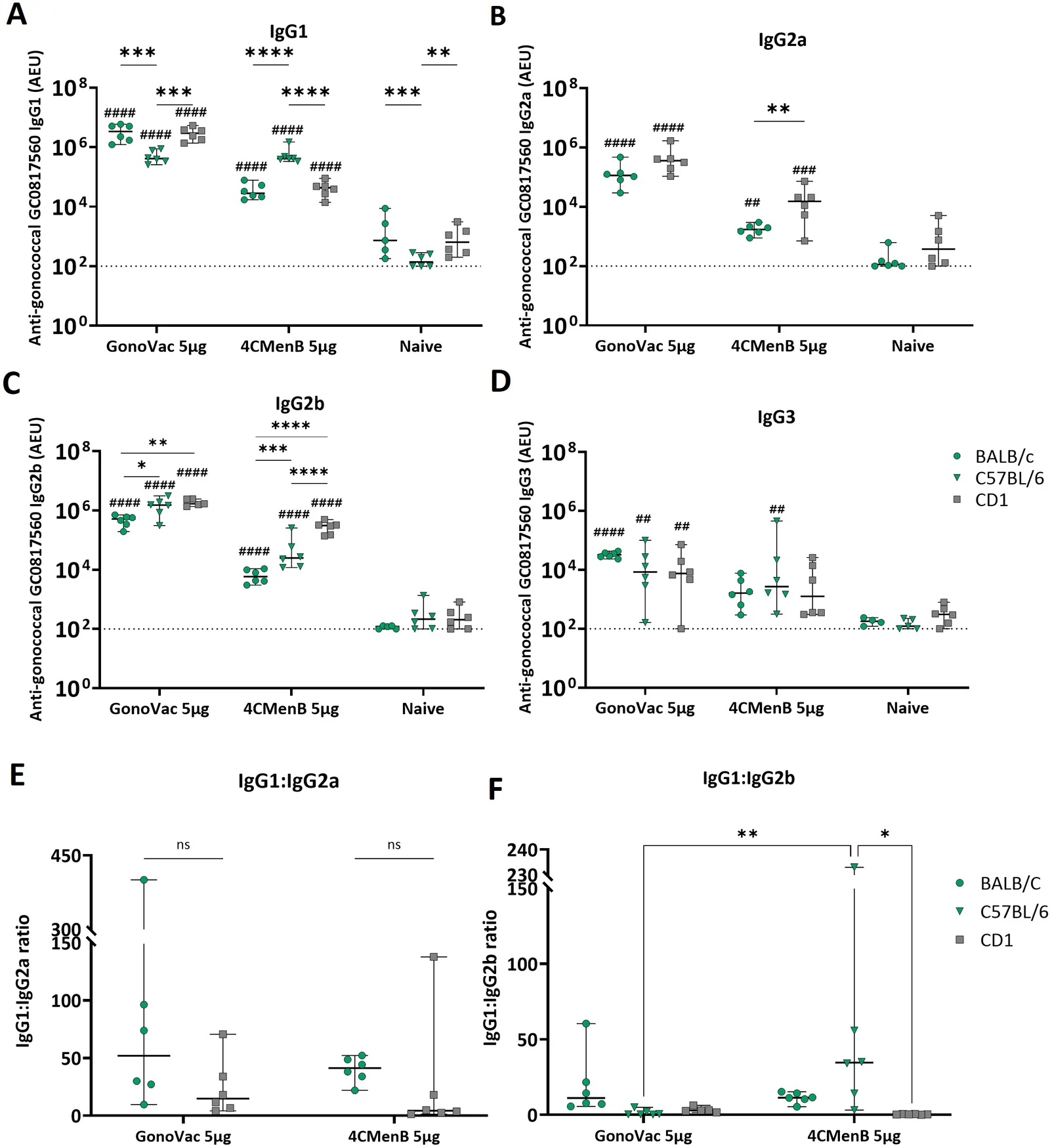
Serum anti-gonococcal IgG subclass distribution in response to immunisation with 3 doses of GonoVac/Al(OH)_3_ or 4CMenB in C57BL/6, BALB/c and CD1 mice at week 9 post 1st dose. 6-8-week-old female mice were immunised intramuscularly at weeks 0, 3 and 6 with 5 µg of flask-produced GonoVac/Al(OH)_3_ or 5 µg of 4CMenB (OMV protein-equivalent). Anti-gonococcal IgG subclass responses were assessed at week 9 by ELISA with *N. gonorrhoeae* GC_0817560 lysate as antigen. Anti-gonococcal **(A)** IgG1, **(B)** IgG2a, **(C)** IgG2b and **(D)** IgG3. **(E)** IgG1/IgG2a and **(F)** IgG1/IgG2b ratio. Statistical test: 2-way ANOVA with Šídák’s correction for multiple comparisons. n=5–6 mice/group. # indicates significant differences relative to the corresponding naïve group. P values: */# p<0.05, **/## p<0.01, ***/### p<0.001, ****/#### p<0.0001. Dotted lines represent the lower limit of quantification (LOQ) for each ELISA, determined as the concentration of the lowest point of the standard curve adjusted by the minimum dilution factor applied to the samples. AEU, Arbitrary ELISA Units.

Vaccine-induced anti-gonococcal serum IgG2a titres tended to be higher in CD1 than BALB/c, with significance seen in mice immunised with 4CMenB (p = 0.007), but not GonoVac (**Figure 2B**). CD1 mice had significantly higher anti-gonococcal IgG2b titres compared with BALB/c following immunisation with GonoVac or 4CMenB, and compared with C57BL/6 mice following immunisation with 4CMenB only (**Figure 2C**).

GonoVac immunisation induced significantly higher levels of all anti-gonococcal IgG isotypes (IgG1, IgG2a, IgG2b, and IgG3) across all mouse strains compared with naïve controls (**Figure 2**; p<0.0001 for IgG1, IgG2a and IgG2b in all strains; IgG3: p = 0.02 in CD1, p = 0.001 in C57BL/6 and p = 0.0001 in BALB/c mice). Similarly, 4CMenB significantly increased anti-gonococcal IgG1, IgG2a, and IgG2b titres in all strains (**Figures 2A–C**; IgG1 and IgG2b: p<0.0001; IgG2a p = 0.002 in CD1 and p = 0.003 in BALB/c mice), although IgG3 responses were only significantly higher than controls in BALB/c mice (**Figure 2D**; p = 0.014).

We assessed the ratio of IgG1:IgG2 subclasses as an indicator of the Th1/Th2 relationship, reflecting the relative contributions of Th2 (IgG1) and Th1 (IgG2) responses to the overall immune response (40). IgG1:IgG2a ratios did not significantly differ between BALB/c and CD1 mice (**Figure 2E**). As C57BL/6 mice do not produce IgG2a, IgG2b was used as a surrogate marker for Th1-associated responses in this strain (41, 42). 4CMenB-immunised C57BL/6 mice had a significantly higher IgG1:IgG2b ratio compared with CD1 mice (p = 0.02, **Figure 2F**).

### 3.3 The ability of GonoVac-induced antibodies to kill *N. gonorrhoeae* along with their avidity persist over time

To assess the long-term functional capacity of vaccine-induced antibodies, we measured antibody-dependent complement-mediated gonococcal killing by SBA at 33 or 49 weeks post-initial immunisation using *N. gonorrhoeae* GC_0817560. A high baseline gonococcal killing was observed in naïve sera of BALB/c mice and some CD1 mice making interpretation of SBA data difficult for these two strains (**Figure 3A**). In contrast, naïve C57BL/6 mice had low baseline SBA titres. At 49 weeks post-immunisation, C57BL/6 mice receiving three doses of GonoVac exhibited significantly higher gonococcal SBA titres compared to both naïve controls (p = 0.02) and 4CMenB-immunised mice (p = 0.009; **Figure 3A**). In CD1 mice, three doses of GonoVac produced significantly higher SBA titres than 4CMenB (p = 0.01; **Figure 3A**). 4CMenB failed to elicit detectable SBA titres in most C57BL/6 and CD1 mice (**Figure 3A**).

**Figure 3:**
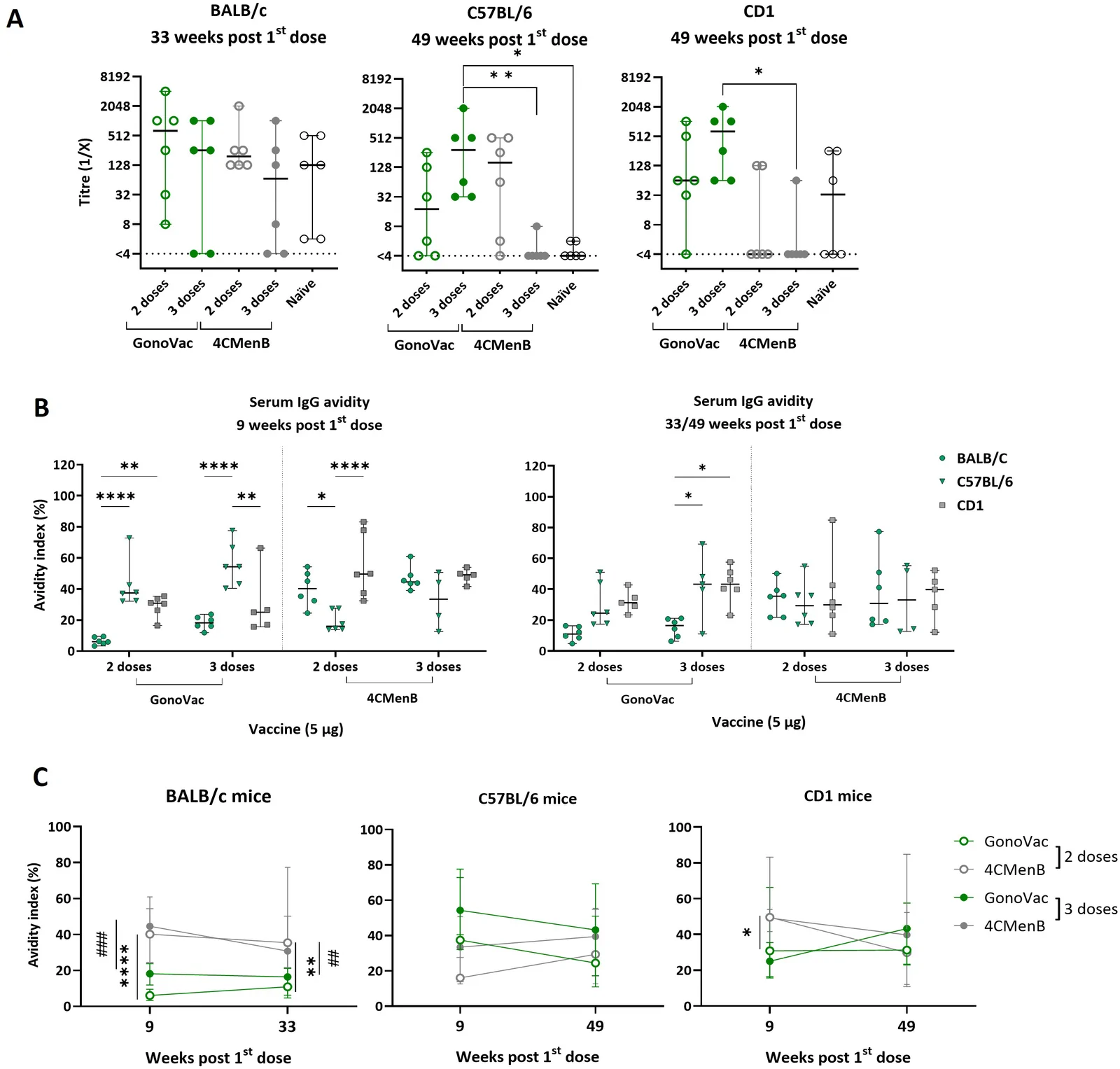
Persistence of serum antibody-dependent complement-mediated killing of *N. gonorrhoeae* and serum anti-gonococcal IgG avidity induced by 2 or 3 doses of GonoVac/Al(OH)_3_ or 4CMenB in C57BL/6, BALB/c and CD1 mice. 6-8-week-old female mice were immunised intramuscularly at weeks 0 and 3 or 0, 3 and 6 with 5 µg of flask-produced GonoVac/Al(OH)_3_ or 5 µg of 4CMenB (OMV protein-equivalent). **(A)** Serum bactericidal assays of sera collected at the end of the study (week 49 or 33) to assess killing of *N. gonorrhoeae* GC_0817560 using 12.5% exogenous complement (IgG/IgM/IgA-depleted human serum). Titres are reciprocals of the last dilution of serum achieving >50% bactericidal killing. Individual mice are represented by circles. Horizontal bars signify medians ± 95% CI. The minimum dilution tested was 1:4. Statistical test: Kruskal-Wallis test with Dunn’s correction for multiple comparisons. **(B)** Serum anti-gonococcal IgG avidity evaluated at week 9 and at the end of the study (week 49 or 33) using ELISA against *N. gonorrhoeae* GC_0817560 lysate with 6M urea used as a chaotropic agent to disrupt low-binding antibodies. Titres obtained after treatment with urea were compared to those obtained without urea to determine the avidity index. Statistical test: 2-way ANOVA with Šídák’s correction for multiple comparisons. **(C)** Median ± 95% CI of avidity response induced by 2 (empty circles) or 3 doses (full circles) between 9- and 33/49-weeks post 1st dose in BALB/c, C57BL/6, and CD1 mice. Statistical differences between GonoVac and 4CMenB-induced IgG avidity are indicated on the sides. Statistical test: 2-way ANOVA with Šídák’s correction for multiple comparisons. n=5–6 mice/group. 2-dose regimen, *; 3-dose regimen, #. P values: */# p<0.05, **/## p<0.01, ***/### p<0.001, ****/#### p<0.0001.

Anti-gonococcal serum IgG avidity was assessed at week 9 and at the study endpoint, revealing distinct avidity profiles for GonoVac and 4CMenB across mouse strains, with avidity numerically lowest in BALB/c and highest in C57BL/6 mice (**Figure 3B**). At week 9, GonoVac-induced IgG avidity was significantly lower in BALB/c than in C57BL/6 (after 2 and 3 doses, p < 0.0001) and CD1 mice (after 2 doses, p = 0.008), while after three immunisations, C57BL/6 mice had significantly higher avidity than CD1 mice, as well as BALB/c mice (p = 0.003 and p<0.0001 respectively; **Figure 3B**). By contrast, 4CMenB-induced avidity was significantly lower after two doses in C57BL/6 mice compared to CD1 (p < 0.0001) and BALB/c (p = 0.01) mice.

By the end of the study, differences in serum anti-gonococcal IgG avidity across mouse strains were less pronounced, with only BALB/c mice, which had received three doses of GonoVac, having significantly lower antibody avidity than C57BL/6 and CD1 mice. No differences were observed in anti-gonococcal IgG avidity between 2 and 3 doses with either vaccine (**Figure 3C**).

Interestingly, in BALB/c mice, 4CMenB elicited higher anti-gonococcal IgG avidity than GonoVac at both time points, irrespective of the number of doses, and higher anti-gonococcal IgG avidity than GonoVac at 9 weeks after 2 doses (**Figure 3C**). The antibody avidity induced by either vaccine did not significantly decline between week 9 to the study endpoint, regardless of mouse strain or number of immunisations (**Figure 3C**).

### 3.4 GonoVac elicits high levels of serum anti-gonococcal IgG antibodies in three strains of mice independent of formulation with Al(OH)_3_, while serum anti-gonococcal IgM levels are higher relative to baseline in BALB/c and C57BL/6 mice following immunisation with GonoVac alone compared with GonoVac/Al(OH)_3_

In a second mouse study, we investigated the impact of Al(OH)_3_ on the immunogenicity GonoVac in BALB/c, C57BL/6, and CD1 mice. Mice received three doses of 5 µg GonoVac (produced in a bioreactor) formulated with or without Al(OH)_3_. Following optimization of antigen adsorption for the bioreactor-produced material, the Al^3+^ content was reduced to 85 µg per 5 µg OMV protein equivalent while maintaining efficient adsorption. Immunisations were administered 3 weeks apart, and blood samples were collected 3 weeks after each immunisation (week 3, 6 and 9) for serological analysis (**Figure 4A**). Additionally, splenocytes and vaginal lavages were collected post-mortem to assess vaccine-induced cellular and mucosal antibody responses.

**Figure 4:**
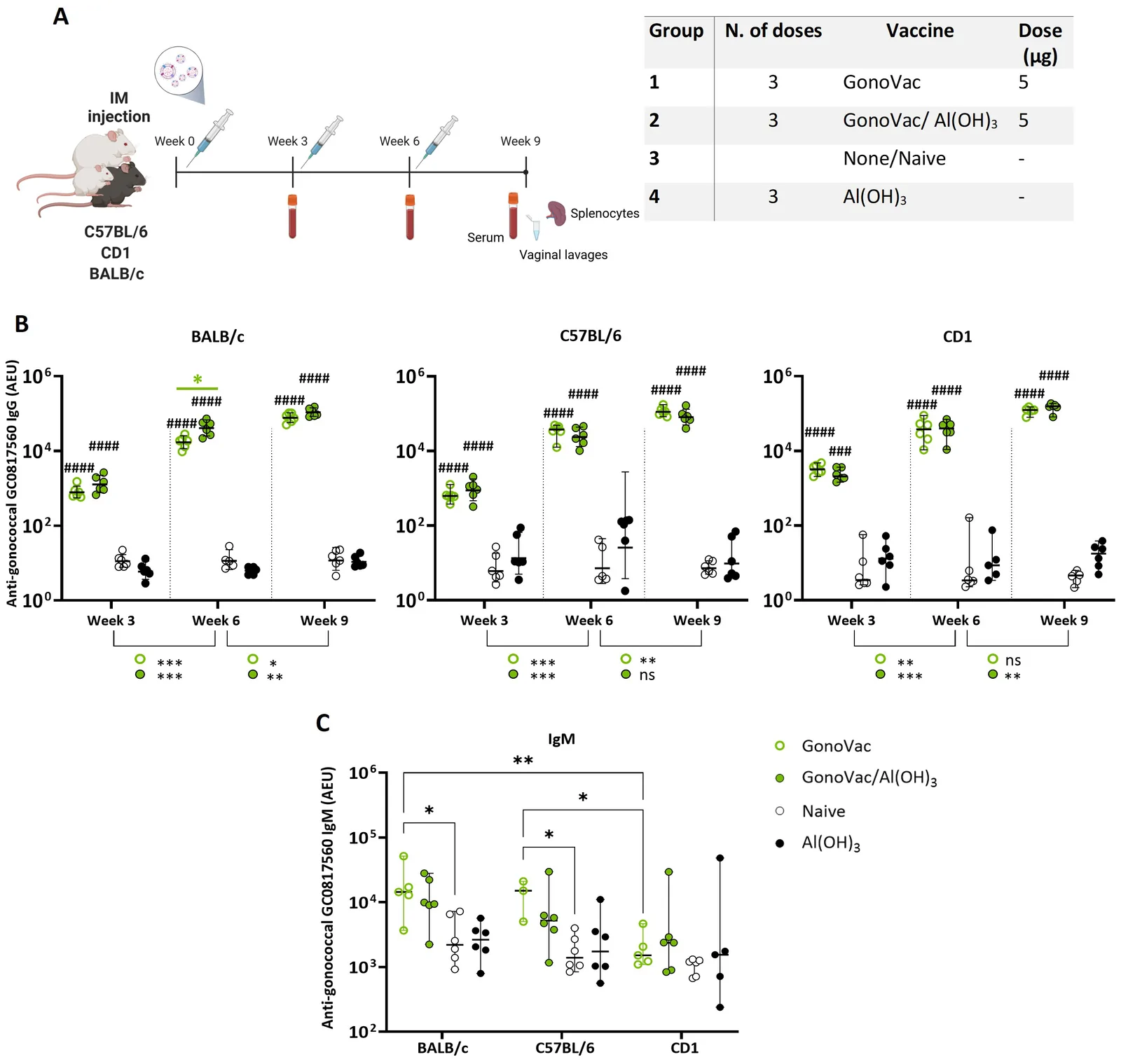
Immunogenicity of GonoVac formulated with and without Al(OH)_3_ in C57BL/6, BALB/c and CD1 mice. **(A)** 6-8-week-old female BALB/c, CD1 and C57BL/6 mice were immunised intramuscularly at weeks 0, 3 and 6 with 5 µg of bioreactor-produced GonoVac formulated with or without Al(OH)_3_. Control groups received either Al(OH)_3_ alone or were not immunised. Serum anti-gonococcal IgG and IgM responses were assessed by ELISA with *N. gonorrhoeae* GC_0817560 lysate as antigen. Figure made with https://www.biorender.com/. **(B)** Serum anti-gonococcal IgG responses evaluated 3 weeks after each immunisation, at week 3, 6 and 9 and **(C)** serum anti-gonococcal IgM at 9 weeks post first immunisation. Statistical test: 2-way ANOVA with Šídák’s correction for multiple comparisons: Individual mice are represented by the circles and horizontal bars signify medians ± 95% CI. n=5–6 mice/group. Significant differences between GonoVac formulated with and without Al(OH)_3_ are indicated by the horizontal green bars and *. # indicates significant differences between GonoVac-immmunised groups, with and without Al(OH)_3_, and their respective negative controls (Al(OH)_3_-only and naïve groups): P values: */# p<0.05, **/## p<0.01, ***/### p<0.001, ****/#### p<0.0001. AEU, Arbitrary ELISA Units.

Following three immunisations, serum anti-gonococcal IgM levels were significantly elevated above controls in BALB/c and C57BL/6 mice immunised with GonoVac alone, but not in mice receiving GonoVac formulated with Al(OH)_3_, indicating a strain-dependent variation in IgM response to GonoVac (**Figure 4B**). CD1 mice produced no detectable IgM following immunisation with either formulation of GonoVac. Notably, there was no statistically significant difference in serum anti-gonococcal IgM levels between mice immunised with GonoVac alone or GonoVac formulated with Al(OH)_3_ for all of mouse strains.

Serum anti-gonococcal IgG levels did not differ significantly between GonoVac formulations in C57BL/6 and CD1 mice. However, in BALB/c mice, GonoVac formulated with Al(OH)_3_ elicited higher serum IgG titres than GonoVac alone (**Figure 4C**), suggesting that the adjuvant enhances anti-gonococcal serum IgG response in this mouse strain. Across all mouse strains, a second immunisation significantly increased serum IgG titres compared with a single dose. A third immunisation further increased serum IgG responses in BALB/c mice irrespective of formulation, in C57BL/6 mice receiving GonoVac without Al(OH)_3_, and in CD1 mice immunised with GonoVac/Al(OH)_3_. (**Figure 4C**).

### 3.5 Formulation with Al(OH)_3_ has little effect on anti-gonococcal IgG subclass antibodies induced by GonoVac

Building on previous observations that serum anti-gonococcal IgG subclass responses to GonoVac vary by mouse strain, we next evaluated the subclass profiles elicited by GonoVac formulated with or without Al(OH)_3_ across the three strains. BALB/c mice immunised with GonoVac without Al(OH)_3_ had significantly higher serum IgG1 titres than C57BL/6 mice (p = 0.047; **Supplementary Figure 1A**). No significant differences were detected in serum anti-gonococcal IgG2a, IgG2b and IgG3 responses between adjuvanted and unadjuvanted GonoVac formulations (**Supplementary Figure 1A**).

Comparing IgG subclass responses across mouse strains, in this mouse study immunised BALB/c mice mounted the highest IgG3 responses, with significantly higher serum anti-gonococcal IgG3 levels than C57BL/6 mice, independent of vaccine formulation (**Supplementary Figure 1A**). As expected IgG1:IgG2a and IgG1:IgG2b ratios, used as an indicator of Th2/Th1 responses, tended to be higher when GonoVac was formulated with Al(OH)_3_ than when administered alone, with significance for IgG1:IgG2a observed in CD1 and for IgG1:IgG2b for BALB/c mice (**Supplementary Figure 1B**). Across mouse strains, IgG1:IgG2b ratio was highest in BALB/c mice and was significantly greater than in C57BL/6 mice following immunisation with either GonoVac alone or GonoVac/Al(OH)_3_, and greater than in CD1 mice when GonoVac was formulated with Al(OH)_3_ (**Supplementary Figure 1B**).

### 3.6 GonoVac induces high levels of antibody-dependent complement-mediated gonococcal killing independent of formulation with Al(OH)_3_ and mouse strain

Following three immunisations with either formulation of GonoVac, all strains of mice had significantly higher antibody-dependent complement-mediated killing of *N. gonorrhoeae* GC_0817560 compared with the respective negative control groups. No significant differences in serum bactericidal activity were observed between mouse strains or GonoVac formulations, indicating that both vaccine formulations effectively induced functional, bactericidal antibody responses irrespective of host genetic background (**Figure 5A**). Despite similar SBA titres, anti-gonococcal serum IgG avidity was higher with GonoVac alone compared with the Al(OH)_3_ formulation in BALB/c mice (**Figure 5B**).

**Figure 5:**
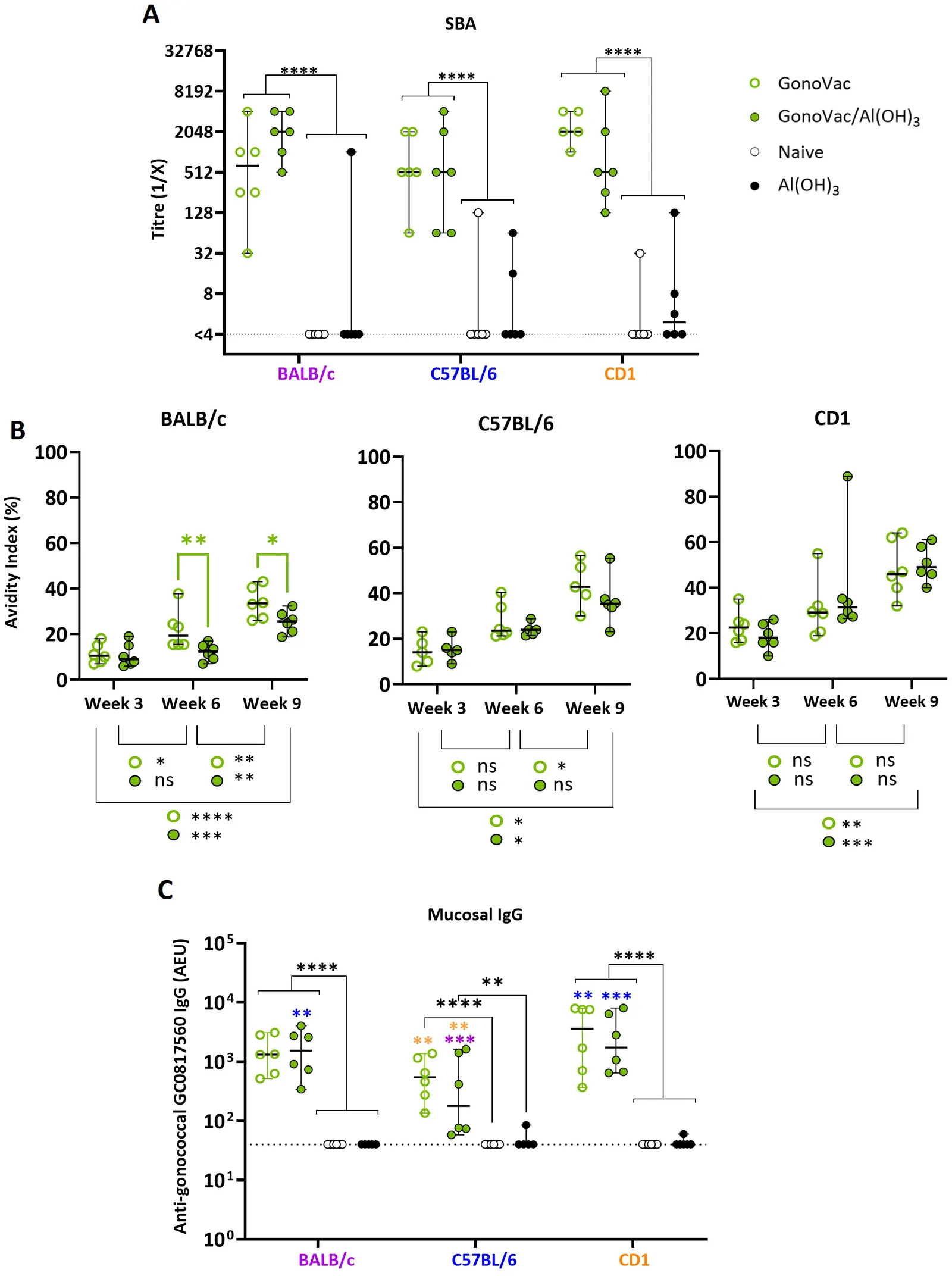
Serum antibody-dependent complement-mediated killing of *N. gonorrhoeae*, anti-gonococcal IgG avidity, and mucosal anti-gonococcal IgG responses induced by GonoVac formulated with and without Al(OH)_3_ in C57BL/6, BALB/c and CD1 mice. 6-8-week-old female BALB/c, CD1, and C57BL/6 mice (6–8 weeks old) were immunised intramuscularly at weeks 0, 3, and 6 with 5 µg of bioreactor-produced GonoVac, formulated with or without Al(OH)_3_. Negative control groups received Al(OH)_3_ alone or were unimmunised. **(A)** Sera collected at the end of the study were analysed by SBA to assess antibody-dependent complement-mediated killing of *N. gonorrhoeae* GC_0817560 using an external source of human complement (12.5% IgG/IgM/IgA-depleted human serum). Titres are reciprocals of the last dilution of serum achieving >50% bactericidal killing. The minimum dilution tested was 1:4. A titre of 1:2 (<4 on the graph) signifies no titre was obtained. **(B)** Serum anti-gonococcal IgG avidity measured after each immunisation using a modified ELISA with 6M urea used as chaotropic agent to disrupt low-binding antibodies. The titres obtained after the treatment with urea were compared to those obtained without urea to determine the avidity index. **(C)** Mucosal anti-gonococcal IgG response measured in vaginal lavages collected post-mortem and quantified using ELISA with *N. gonorrhoeae* GC_0817560 lysate as antigen. Dotted line represents the lower limit of quantification (LOQ) of the ELISA, determined as the concentration of the lowest point of the standard curve adjusted by the minimum dilution factor applied to the samples. Statistical test: 2-way ANOVA with Šídák’s correction for multiple comparisons. P values: *p < 0.05; **p < 0.01; ***p < 0.001; ****p < 0.0001. Each circle represents an individual mouse. n = 5–6/group. Horizontal bars represent medians ± 95% CI. Significant differences between mouse strains are indicated in the respective strain colours. AEU, Arbitrary ELISA Units.

Serum anti-gonococcal IgG avidity maturation was observed across all strains following repeated immunisations, supporting a role of more than one vaccine dose in improving antibody quality (**Figure 5B**). Across all mouse strains tested, a significant increase in serum IgG avidity was consistently evident following three immunisations.

### 3.7 GonoVac induces mucosal IgG particularly in outbred CD1 mice irrespective of formulation with Al(OH)_3_

To gain insight into vaccine-induced immunity at the mucosal surface, anti-gonococcal IgG levels were measured in vaginal washes collected after three immunisations. Both GonoVac formulations elicited significantly higher mucosal anti-gonococcal IgG responses compared with controls, regardless of mouse strain (**Figure 5C**). While no significant differences were observed between the adjuvanted and non-adjuvanted groups, strain-dependent variation was apparent with CD1 and BALB/c mice having higher vaginal IgG titres than C57BL/6 mice.

### 3.8 Cellular immune responses to GonoVac are mouse strain- and formulation-dependent

The cellular immune response to GonoVac, assessed via FluoroSpot assay, revealed notable formulation- and strain-dependent differences in levels of gonococcal-specific cytokine-producing splenocytes. In BALB/c mice, immunisation with GonoVac formulated with Al(OH)_3_ elicited significantly higher numbers of gonococcal-specific IFN-γ and IL-17A spot-forming cells (SFCs) compared with both GonoVac-alone and negative control groups, indicating robust Th1 and Th17 responses associated with formulation with aluminium-containing adjuvant. Following immunisation with GonoVac/Al(OH)_3_, the levels of cells producing IFN-γ and IL-17A in BALB/c mice in response to stimulation with gonococcal lysate were higher than in both C57BL/6 and CD1 strains (p<0.0001) (**Figures 6A, C**).

**Figure 6:**
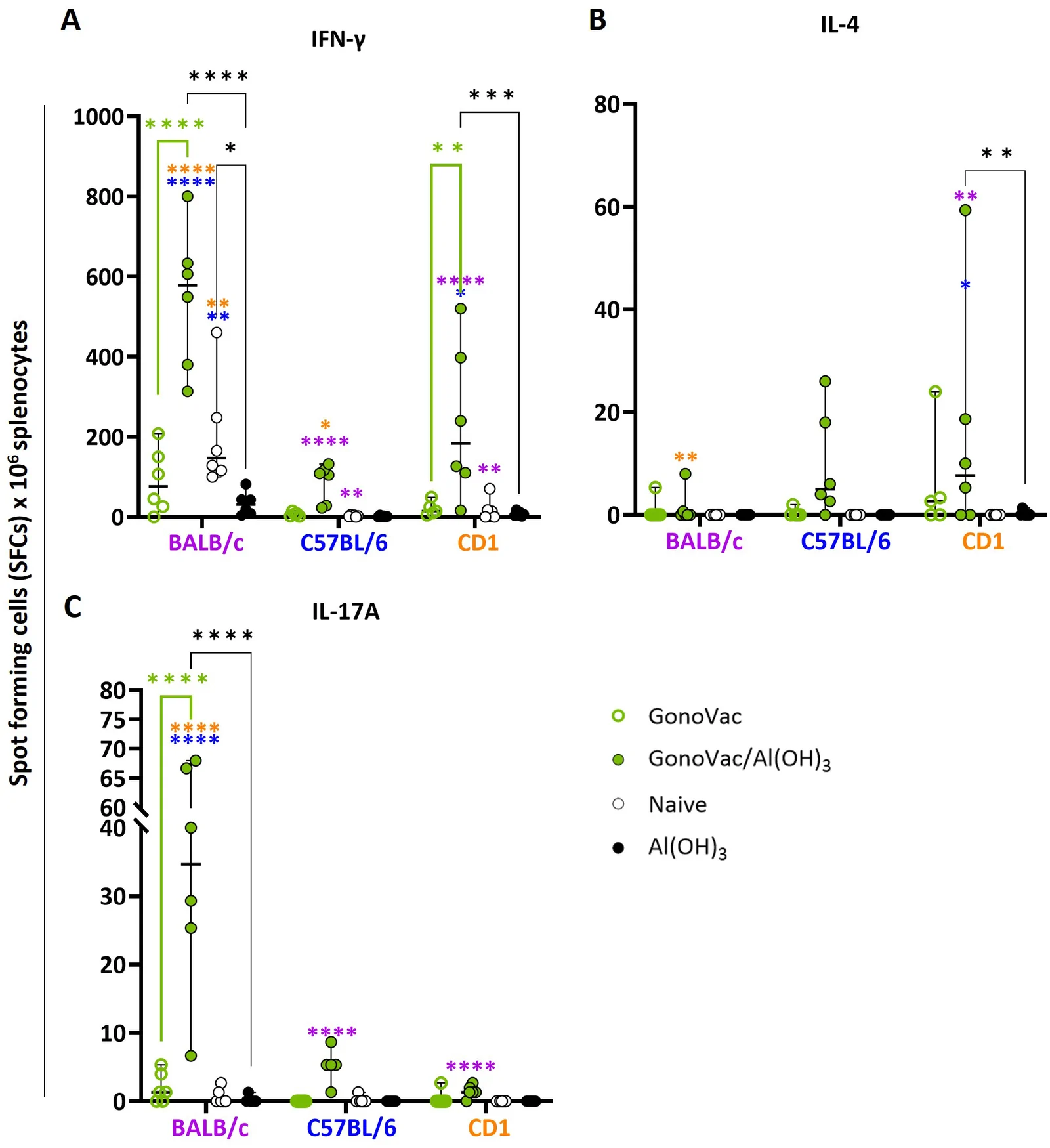
Cellular immune responses induced by GonoVac formulated with and without Al(OH)_3_ in C57BL/6, BALB/c and CD1 mice. 6-8-week-old female BALB/c, CD1, and C57BL/6 mice were immunised intramuscularly at weeks 0, 3, and 6 with 5 µg of bioreactor-produced GonoVac, formulated with or without Al(OH)_3_. Negative control groups received Al(OH)_3_ alone or were unimmunised. Splenocytes collected post-mortem were restimulated ex vivo with *N. gonorrhoeae* GC_0817560 lysate to assess the levels of **(A)** IFN-γ-; **(B)** IL-4- and **(C)** IL-17A-producing splenocytes. The number of spot-forming cells (SFCs) is shown after correction for background (unstimulated controls). Statistical test: 2-way ANOVA with Šídák’s correction for multiple comparisons. P values: *p < 0.05; **p < 0.01; ***p < 0.001; ****p < 0.0001. Each circle represents an individual mouse. n = 5–6/group). Horizontal bars represent medians ± 95% CI. Significant differences between mouse strains are indicated in the respective strain colours.

Unimmunised BALB/c mice had high levels of gonococcal-specific IFN-γ-, but not IL-4- or IL-17A-producing cells (**Figures 6A, B**). Similar to BALB/c mice, CD1 mice immunised with GonoVac formulated with Al(OH)_3_ exhibited significantly higher gonococcal-specific IFN-γ-producing cell frequencies compared to non-immunised controls and the GonoVac-alone group (**Figure 6A**). Gonococcal-specific IL-4-producing cells remained low across all groups, with the only significant increase observed in CD1 mice immunised with GonoVac/Al(OH)_3_ (**Figure 6B**).

## 4 Discussion

The increasing global incidence of gonorrhoea, especially in LMICs, exacerbated by the emergence of multidrug-resistant strains and the threat of untreatable gonorrhoea, underscores the urgent need for an effective vaccine. Current preclinical strategies include single- and multi-antigen approaches such as those targeting the gonococcal 2C7 lipooligosaccharide epitope (43) or different variants of transferrin binding protein B (44), mucosal delivery of gonococcal OMVs with microencapsulated IL-12 (45, 46), modified meningococcal OMVs for pan-*Neisseria* protection (47, 48), and gonococcal-specific OMV formulations (23, 24, 49). Recent randomised controlled trials evaluating 4CMenB against gonorrhoea, including the DOXYVAC (50), and GoGoVax trials (51), failed to demonstrate significant protection in high-risk populations, highlighting the need for gonococcus-specific vaccine approaches.

In response to these challenges, we are developing GonoVac, a gonococcal native OMV candidate vaccine specifically designed to induce durable, targeted protective immunity against *N. gonorrhoeae* at low cost of goods (23, 24). In the present work, in a first mouse study, we evaluated the long-term immunogenicity of GonoVac across three genetically distinct strains of mouse and compared immune responses with those induced by 4CMenB. In a second mouse study we explored the immunomodulatory effects of Al(OH)_3_ by comparing GonoVac alone with GonoVac formulated with Al(OH)_3_, assessing the resulting humoral and cellular acquired immune responses.

Our findings demonstrate that GonoVac/Al(OH)_3_ induces robust, long-lasting anti-gonococcal IgG responses across three commonly used laboratory mouse strains, BALB/c, C57BL/6 and CD1, with titres remaining elevated up to a year post-immunisation, irrespective of whether given as a two-dose or three-dose regime. With outbred CD1 mice, perhaps most closely resembling the genetic heterogeneity of humans, and inbred BALB/c mice, there was no difference in anti-gonococcal IgG titres at one year compared with levels at nine weeks, three or six weeks after two doses or three doses of GonoVac/Al(OH)_3_. This is important as a successful gonococcal vaccine will need to induce durable as well as protective immunity (37, 52). However, in CD1 mice immunised with 4CMenB there was a significant decline in anti-gonococcal IgG levels over time following immunisation.

When combined with the higher initial anti-gonococcal IgG levels induced by GonoVac compared with 4CMenB, the longevity of the immune response to GonoVac indicates potential superior capacity of GonoVac over 4CMenB to sustain long-term protective antibody responses across genetically diverse populations. Such durability could be particularly relevant given the need for sustained protection against gonorrhoea in light of the high rates of reinfection observed in high-risk groups and the need to protect adolescents from sexual debut and throughout adulthood. Both populations have been identified as priority target groups for gonococcal vaccination (37, 53). The New Zealand MeNZB study which first identified the potential of meningococcal B vaccines to protect against gonorrhoea found that protection waned after two years (10).

Distinct immunophenotypic signatures were observed between GonoVac vaccine formulations (with and without Al(OH)_3_) and across mouse strains. In the first study, GonoVac induced superior and durable anti-gonococcal functional humoral immunity in C57BL/6 mice, evidenced by significantly higher levels of antibody-dependent complement-mediated killing of *N. gonorrhoeae* compared with 4CMenB. However, high background SBA titres made findings difficult to interpret among BALB/c and CD1 mice in this study. This background likely reflects naturally occurring cross-reactive IgM antibodies capable of mediating complement-dependent bactericidal activity against *N. gonorrhoeae* as previously described (54–57). However, in the second mouse study, minimal baseline SBA titres allowed us to demonstrate highly significant antibody-dependent complement-mediated killing of *N. gonorrhoeae* for all three mouse strains at 9 weeks following immunisation with three doses of GonoVac.

In contrast to GonoVac, anti-gonococcal serum IgG induced by 4CMenB lacked corresponding bactericidal function, despite demonstrating ability to bind gonococcal antigen and high avidity. We speculate that reason for this disconnect between antibody binding and functional activity is the result of 4CMenB lacking critical and, as yet, unidentified, gonococcal antigens that are key targets of bactericidal antibodies. Alternatively, 4CMenB-induced antibodies could predominantly target immune-evasive or non-protective antigens such as Rmp (58, 59), which shares more than 94% sequence homology with its meningococcal ortholog (60) and is present in the detergent-extracted OMVs of the New Zealand MenB strain used in 4CMenB, as well as GonoVac. It is possible that differences in 4CMenB- compared with GonoVac-induced anti-gonococcal IgG subclass distribution or Fc glycosylation could impair complement engagement and killing of gonococci (61–64).

The effects of formulation with Al(OH)_3_ on GonoVac immunogenicity are complex and appeared highly dependent on host genetic background. In addition, GonoVac formulations used in the longevity and formulation comparison studies differed. In the longevity study, GonoVac was produced in shake flasks and formulated with 259.5 µg Al^3+^ per 5 µg antigen, whereas in the formulation comparison study, which took place after optimisation of antigen adsorption with bioreactor-produced material, the aluminium content was reduced to 85 µg Al^3+^ per 5 µg antigen, an amount that retained efficient OMV adsorption. Thus, comparison between the two studies needs to consider differences in both OMV production method and Al^3+^ to antigen ratio.

Previously, we had demonstrated in BALB/c mice that formulation of GonoVac with Al(OH)_3_ significantly enhanced serum anti-gonococcal IgG responses (24). However, in the present work this effect was found to be strain-dependent, as similar increases were not observed in C57BL/6 and CD1 mice, suggesting that the adjuvant effects of Al(OH)_3_ are more pronounced in inbred Th2-prone animals, such as BALB/c mice, and not in outbred populations such as CD1 mice and humans.

Although both GonoVac formulations induced comparable SBA titres, which did not vary across strains of mouse, suggesting similar induction of complement-activating antibodies, differences became apparent in antibody avidity maturation. Serum anti-gonococcal IgG avidity was significantly higher in BALB/c mice immunised with GonoVac formulated without Al(OH)_3_ after two and three doses with avidity progressively increasing following each dose. Across all mouse strains tested, a significant increase in serum IgG avidity was evident after three doses of GonoVac, with or without Al(OH)_3_. These findings align with our previous findings in BALB/c mice (24) and suggest that while aluminium adjuvant enhances some aspects of acquired immunity, it may also modulate or delay affinity maturation in certain genetic backgrounds. Similar phenomena have been reported for other OMV-based vaccines; for instance, unadjuvanted *Shigella sonnei* GMMA vaccines induced higher SBA titres than *S. sonnei* GMMA formulated with Al(OH)_3_ or with TLR7/8 agonists (65). Collectively, these results suggest that although Al(OH)_3_ does not impair the induction of GonoVac-induced bactericidal antibodies, it may influence the kinetics of antibody maturation, with increases in avidity only emerging following repeated immunisation.

Consistent with our previous observations in BALB/c mice (24) systemic immunisation with GonoVac induced anti-gonococcal mucosal IgG responses across all mouse strains tested, independent of Al(OH)_3_ formulation. However, the magnitude of these responses varied between strains, suggesting an influence of host genetic background. In humans, IgG is the dominant immunoglobulin at type II mucosal surfaces, such as the vaginal tract, where it is actively transported from circulation into the lumen via the neonatal Fc receptor (FcRn) (66). Once at the mucosal surface, IgG mediates protection by blocking bacterial adherence, neutralising pathogens, and promoting phagocytosis and complement activation (67). Thus, the capacity of GonoVac to elicit robust mucosal IgG responses via systemic immunisation supports its potential as a promising vaccine candidate against this mucosally transmitted pathogen. However, as with other immune parameters assessed in the current study, mucosal anti-gonococcal IgG is not a correlate of protection against gonorrhoea.

Despite relatively modest effects on acquired humoral immunity, formulation of GonoVac with Al(OH)_3_ significantly improved vaccine-induced Th1- and Th17-associated cellular immune responses in BALB/c mice, as we previously reported (24). However, cellular immune responses in other mouse strains were muted, particularly in C57BL/6 mice where there was a relative absence of gonococcal-specific IFNƳ-, IL-4- and IL-17A-secreting splenocytes. This is perhaps surprisingly given C57BL/6 mice are predisposed towards strong Th1 responses and IFN-γ production (25, 32–34). Furthermore, our findings suggest that the immunomodulatory effects of Al(OH)_3_ vary according to host genetic background. Somewhat paradoxically, despite the well-established Th2 predisposition of BALB/c mice, stimulation of splenocytes with gonococcal lysate induced high levels of IFN-γ- but not IL-4-producing cells in immunised BALB/c mice. This unexpected Th1-type cytokine profile may reflect the high TLR4 expression and responsiveness in BALB/c immune cells (68), considering gonococcal lysate used for stimulation contains intact lipid A, a potent TLR4 agonist (69, 70), capable of inducing strong innate TLR4-mediated activation.

The observed pattern of cytokine-secreting splenocytes is not without precedent: BALB/c mice immunised with BCG mount stronger Th1 and Th17 responses than C57BL/6 mice, including higher frequencies of IFN-γ-, IL-2- and IL-17-producing cells, while C57BL/6 mice instead favor IL-10 production (71), indicating that strain-associated T-helper bias can depend on the nature of the antigen and immunological stimulus rather than being a fixed, intrinsic property of the mouse strain. Although aluminium salts have classically been regarded as Th2-promoting adjuvants, this view is largely derived from studies using purified protein antigens lacking PAMPs. Co-delivery of aluminium salts with TLR ligands has by contrast been shown to promote balanced Th1/Th2 or Th1-skewed responses (72). It is therefore plausible that the combined effects of endogenous OMV-derived TLR agonists, formulation with Al(OH)_3_ and innate immune activation contributed to the elevated IFN-γ responses in Al(OH)_3_-adjuvanted BALB/c mice.

Although GonoVac protective efficacy was not directly assessed as part of the present work, the acquired immune responses described may reflect mechanisms contributing to previously reported gonococcal OMV-mediated protection by us and others (23, 45). Murine immune responses do not fully recapitulate those observed in humans, and additional immune mechanisms, including opsonophagocytic and mucosal antibody function, were not evaluated in the current work, limiting conclusions regarding correlates of protection. Furthermore, the immune cell populations underlying the cytokine responses are yet to be defined. Future studies of GonoVac should investigate the protective efficacy and associated immune responses in preclinical and clinical gonococcal challenge models, where delineation of efficacy can help define correlates of protection.

As part of future work, we need to assess the breadth of vaccine-induced immune responses against a collection of genetically diverse gonococcal strains. It will also be valuable to assess the immunogenicity GonoVac in male as well as female mice. As we have recently demonstrated the efficacy of gonococcal OMV vaccines against *N. gonorrhoeae* infection in the genital tract of female mice (23), we restricted the current study to female mice in order to investigate more specifically immune responses that might underlie this efficacy. We did not assess IgA responses as we previously found that GonoVac induces minimal serum IgA and very little secretory IgA (24).

In conclusion, we have systematically compared the durability of immune responses induced by a gonococcal nOMV candidate vaccine across multiple mouse genetic backgrounds over a one-year period. GonoVac induces strong, long-lasting acquired functional systemic and mucosal humoral immune responses with minimal variability across mouse strains, generating anti-gonococcal immune responses that are both more durable and of greater magnitude than those elicited by 4CMenB. We have also assessed the immunogenicity of GonoVac with and without formulation with Al(OH)_3_ across these mouse strains and found limited differences in the humoral immune responses elicited and enhanced cellular immunity in BALB/c and CD1 mice when formulated with Al(OH)_3_ but not in C57BL/6 mice. Our findings support the potential of GonoVac as a broadly immunogenic candidate vaccine across different genetic backgrounds of host.

## Data Availability

The raw data supporting the conclusions of this article will be made available by the authors, without undue reservation.
