# Supplementary material for: A candidate gonococcal outer membrane vesicle vaccine which induces persistent immunity in three genetically distinct species of mouse and is similarly immunogenic when formulated with and without aluminium adjuvant
Source: Front Immunol. 2026 Oct 2;17:1923399. doi: 10.3389/fimmu.2026.1923399 (PMC13672624; doi:10.3389/fimmu.2026.1923399)
Supplement: Supplementary file 1 [file DataSheet1.docx]

Supplementary Material

# 1 Supplementary Figures and Tables

**Supplementary Figure 1:** **Serum anti-gonococcal IgG subclass distribution induced by GonoVac formulated with or without Al(OH)_3_ in genetically distinct mouse strains.** Female BALB/c, C57BL/6 and CD1 mice (6–8 weeks old) were immunised intramuscularly at weeks 0, 3, and 6 with 5 µg of bioreactor-produced GonoVac, formulated with or without Al(OH)_3_. Negative control groups received Al(OH)₃ alone or were unimmunised.

(A) Serum samples collected at the end of the study were analysed by ELISA to assess anti-gonococcal IgG subclass distribution, using *N. gonorrhoeae* GC_0817560 lysate as antigen. Results are expressed in Arbitrary ELISA Units (AEU). The dotted lines represent the lower limit of quantification (LOQ) for each ELISA assay, determined as the concentration of the lowest point of the standard curve adjusted by the minimum dilution factor applied to the samples.

(B) Serum IgG1:IgG2a and IgG1:IgG2b ratios were calculated as indicators of Th2/Th1 responses.

Statistical test: 2-way ANOVA with Šídák’s correction for multiple comparisons. P values: *p < 0.05; **p < 0.01; ***p < 0.001; ****p < 0.0001. Each circle represents an individual mouse (n = 5–6 per group); data are presented as median ± 95% CI. Significant differences between mouse strains are indicated in the respective strain colours.
